# Persistent fasting lipogenesis links impaired ketogenesis with citrate synthesis in humans with nonalcoholic fatty liver

**DOI:** 10.1172/JCI167442

**Published:** 2023-05-01

**Authors:** Xiaorong Fu, Justin A. Fletcher, Stanisław Deja, Melissa Inigo-Vollmer, Shawn C. Burgess, Jeffrey D. Browning

**Affiliations:** 1 Center for Human Nutrition,; 2 Department of Clinical Nutrition,; 3 Department of Biochemistry,; 4 Department of Pharmacology, and; 5 Department of Internal Medicine, University of Texas Southwestern Medical Center at Dallas, Dallas, Texas, USA.

**Keywords:** Endocrinology, Hepatology, Fatty acid oxidation, Intermediary metabolism

## Abstract

**BACKGROUND:**

Hepatic de novo lipogenesis (DNL) and β-oxidation are tightly coordinated, and their dysregulation is thought to contribute to the pathogenesis of nonalcoholic fatty liver (NAFL). Fasting normally relaxes DNL-mediated inhibition of hepatic β-oxidation, dramatically increasing ketogenesis and decreasing reliance on the TCA cycle. Thus, we tested whether aberrant oxidative metabolism in fasting NAFL subjects is related to the inability to halt fasting DNL.

**METHODS:**

Forty consecutive nondiabetic individuals with and without a history of NAFL were recruited for this observational study. After phenotyping, subjects fasted for 24 hours, and hepatic metabolism was interrogated using a combination of ^2^H_2_O and ^13^C tracers, magnetic resonance spectroscopy, and high-resolution mass spectrometry.

**RESULTS:**

Within a subset of subjects, DNL was detectable after a 24-hour fast and was more prominent in those with NAFL, though it was poorly correlated with steatosis. However, fasting DNL negatively correlated with hepatic β-oxidation and ketogenesis and positively correlated with citrate synthesis. Subjects with NAFL but undetectable fasting DNL (25th percentile) were comparatively normal. However, those with the highest fasting DNL (75th percentile) were intransigent to the effects of fasting on the concentration of insulin, non-esterified fatty acid, and ketones. Additionally, they sustained glycogenolysis and were spared the loss of oxaloacetate to gluconeogenesis in favor of citrate synthesis, which correlated with DNL and diminished ketogenesis.

**CONCLUSION:**

Metabolic flux analysis in fasted subjects indicates that shared metabolic mechanisms link the dysregulations of hepatic DNL, ketogenesis, and the TCA cycle in NAFL.

**TRIAL REGISTRATION:**

Data were obtained during the enrollment/non-intervention phase of Effect of Vitamin E on Non-Alcoholic Fatty Liver Disease, ClinicalTrials.gov NCT02690792.

**FUNDING:**

This work was supported by the University of Texas Southwestern NORC Quantitative Metabolism Core (NIH P30DK127984), the NIH/National Institute of Diabetes and Digestive and Kidney Diseases (R01DK078184, R01DK128168, R01DK087977, R01DK132254, and K01DK133630), the NIH/National Institute on Alcohol Abuse and Alcoholism (K01AA030327), and the Robert A. Welch Foundation (I-1804).

## Graphical abstract

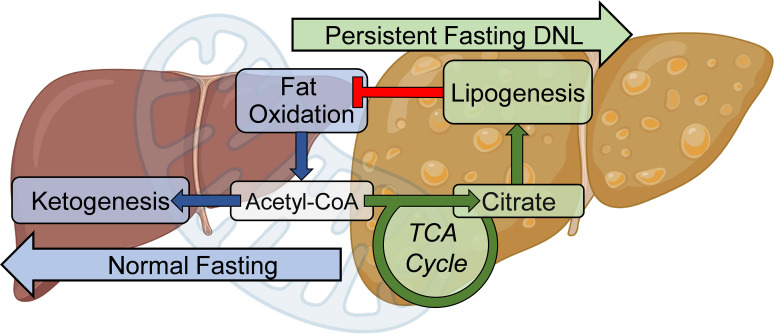

## Introduction

Nonalcoholic fatty liver (NAFL) is a highly prevalent condition associated with obesity and insulin resistance that, in some individuals, will progress to nonalcoholic steatohepatitis (NASH), cirrhosis, and liver failure (1, 2). Hepatic steatosis is the primary feature of NAFL, defined as the accumulation of triglyceride (TG) greater than 2%–5% of liver volume (1, 3, 4). Tracer studies indicate that excess hepatic TG in NAFL originates from elevated de novo lipogenesis (DNL) (5–9) and the influx of non-esterified fatty acid (NEFA) released from insulin-resistant adipose tissue (10, 11). In addition, before the onset of NASH, lipid accrual is partially compensated by increased oxidative metabolism (10, 12, 13), with increased mitochondrial reactive oxygen species (ROS) production (14) — a process thought to contribute to hepatocellular damage and disease progression (2, 15, 16). Dysregulation of both hepatic lipid synthesis and mitochondrial function are widely accepted factors in NAFL, but whether they share metabolic mechanisms in humans is unclear.

Excess DNL during NAFL is expected to inhibit mitochondrial β-oxidation. The conversion of cytosolic acetyl-CoA to malonyl-CoA by acetyl-CoA carboxylase (ACC) is the first committed step in DNL. In turn, malonyl-CoA reciprocally opposes mitochondrial β-oxidation by allosteric inhibition of carnitine acyltransferase (CPT-1) transport of NEFA (17, 18) (Supplemental Figure 1; supplemental material available online with this article; https://doi.org/10.1172/JCI167442DS1). However, tracer studies indicate that NEFA oxidation in the hepatic tricarboxylic acid (TCA) cycle is normal in lean NAFL subjects (19) and increased in obese subjects with NAFL (10, 20–23), findings that parallel studies of ex vivo mitochondrial respiration (12, 14). To challenge the capacity of in vivo hepatic fat oxidation, we recently fasted subjects for 24 hours, which induced β-oxidation more than 2-fold (21) in comparison with overnight-fasted subjects (10) (Supplemental Figure 1), consistent with suppression of DNL and malonyl-CoA levels. NAFL subjects induced β-oxidation during fasting, but they had lower rates of ketogenesis and an increased reliance on the TCA cycle (21). It is unknown whether this oxidative shift originates from mitochondrial dysfunction or whether it is metabolically mediated.

The partitioning of acetyl-CoA between ketogenesis and the TCA cycle is metabolically determined by the rate of β-oxidation, availability of free CoA, and the capacity of the TCA cycle to accept acetyl-CoA (24, 25) (Supplemental Figure 1). During fasting, increased circulating NEFA and low DNL/malonyl-CoA provoke high rates of acetyl-CoA production by β-oxidation. However, fasting also results in increased hepatic gluconeogenesis, diminishing anaplerotic substrate (e.g., lactate, pyruvate, and amino acids), and a more reduced redox state, factors that deplete the TCA cycle of oxaloacetate and limit citrate synthesis (24, 26). Activation of ketogenesis provides the periphery with an alternative substrate to dwindling glucose availability, consumes acetyl-CoA, recovers free CoA necessary for accelerated fatty acid processing, and can support an order of magnitude higher rate of β-oxidation than the TCA cycle alone (24, 25).

In contrast, TCA cycle intermediates are replete in the postprandial state owing to abundant anaplerotic substrate, and they are further spared by ample glycogen and decreased loss to gluconeogenesis. A plentiful supply of oxaloacetate facilitates synthesis of citrate and its oxidation in the TCA cycle (26, 27), which results in increased ATP yield in comparison with β-oxidation linked to ketogenesis. When cellular ATP demand is met, excess citrate is transported to the cytosol, where ATP citrate lyase regenerates acetyl-CoA to synthesize malonyl-CoA by ACC, promoting DNL and concomitantly suppressing mitochondrial β-oxidation. Perhaps for this reason, low-carbohydrate diets and ACC inhibitors, which suppress DNL and enhance ketogenesis, are very effective at reducing hepatic steatosis (28–32).

Obese NAFL subjects have elevated ketones in postabsorptive studies, consistent with increases in lipolysis, circulating NEFA, and hepatic β-oxidation. However, fasting ketosis is blunted in several studies, suggesting limited β-oxidation capacity (15). Therefore, we hypothesized that sustained TCA cycle metabolism and suppressed ketogenesis in fasted NAFL subjects (21) reflect elevated hepatic malonyl-CoA in these individuals. Since hepatic malonyl-CoA cannot be noninvasively assessed in human liver, we used an ultrasensitive high-resolution Orbitrap mass spectrometry (MS) method (33) to assess fasting DNL as a surrogate of malonyl-CoA synthesis. We report that, in previously characterized NAFL subjects (21), persistent fasting DNL links decreased β-oxidation and ketogenesis to an increased reliance on the TCA cycle.

## Results

### NAFL subjects fail to suppress DNL completely during fasting.

**Figure 1:**
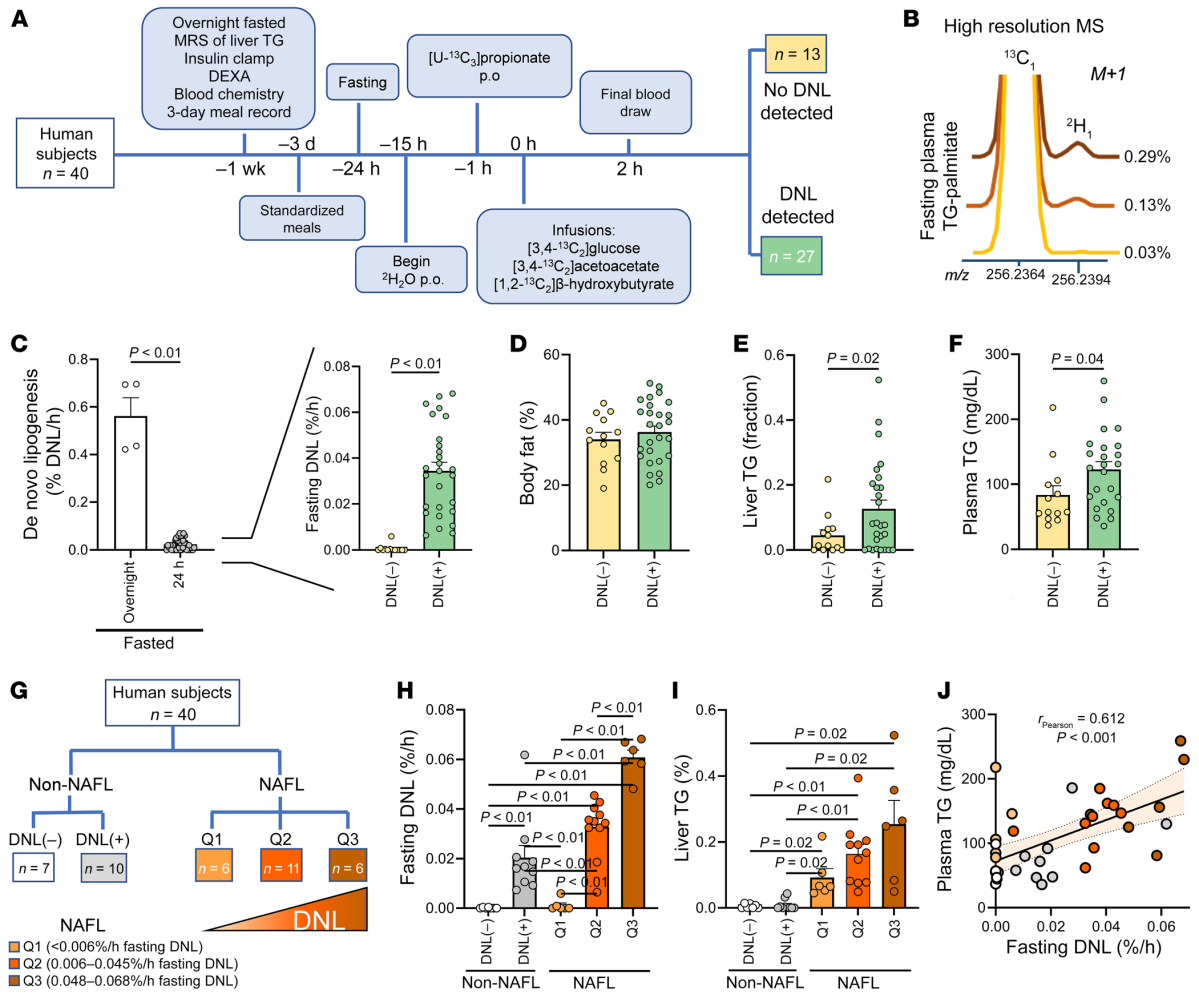
DNL persists after a 24-hour fast in some subjects. (**A**) Study schema. (**B**) High-resolution Orbitrap gas chromatography–mass spectrometry (MS) of palmitate isolated from plasma TG of humans with various degrees of fasting DNL. The ^2^H M+1 enrichment was easily resolved from the M+1 of ^13^C and was used to determine DNL. (**C**) Fractional DNL in overnight- (*n* = 4) and 24-hour-fasted (*n* = 40) subjects. (**D**) Percentage body fat, measured by DEXA, in DNL(–) (*n* = 13) and DNL(+) (*n* = 27) subjects. (**E**) Liver triglyceride (TG) measured by ^1^H-MRS. (**F**) Plasma TGs. (**G** and **H**) Subjects without NAFL (Non-NAFL) were dichotomized by the presence or absence of persistent fasting DNL. NAFL subjects were ranked according to fasting DNL activity and divided into quartiles (Q1, lower 25th percentile; Q2, middle 25th to 75th percentile; Q3, upper 75th percentile). (**I**) Hepatic TG content across the range of fasting DNL. (**J**) Plasma TG across the spectrum of fasting DNL. Statistical significance was determined by 2-tailed *t* test, 1-way ANOVA to analyze multiple groups, and Pearson correlation. Non-correlative data are presented as mean ± SEM.

Subjects (*n* = 40) were previously characterized over the course of 2 weeks and received a 3-day standardized diet before a 24-hour fast (21) (Figure 1A). Subject characteristics are presented in Table 1. They consumed ^2^H_2_O in divided doses beginning the night before the study, resulting in an average body water enrichment of 0.52% (range 0.39%–0.65%). After a 24-hour fast, subjects received ^13^C tracers to assess glucose synthesis, hepatic TCA cycle metabolism, and ketogenesis, previously collated according to liver steatosis (21). Plasma was collected following tracer infusions, and TG was extracted, hydrolyzed, and analyzed for 24-hour fasted palmitate ^2^H enrichment by high-resolution Orbitrap MS. The palmitate M+1 isotopologue of ^2^H was easily resolved from the natural abundance M+1 of ^13^C (Figure 1B). Subjects with greater than 0.10% palmitate ^2^H enrichment were considered to have detectable DNL (33). DNL was approximately 20-fold lower after a 24-hour fast than after an overnight fast but could be detected in 68% of subjects (Figure 1C). This persistent fasting DNL was independent of adiposity (Figure 1D). However, subjects with detectable DNL had higher liver TG content (Figure 1E) and increased plasma TGs (Figure 1F). Interestingly, many lipogenic subjects were non-obese with no evidence of impaired fasting glucose or fatty liver.

To clarify the differential effects of hepatic steatosis, non-NAFL subjects (liver TG <5%) were classified as DNL(–) (*n* = 7) or DNL(+) (*n* = 10), and NAFL subjects (liver TG ≥5%) were ranked according to fasting DNL activity (Figure 1G and Table 2). The lower quartile of DNL in NAFL subjects (*n* = 6, Q1) had undetectable fasting DNL, similar to non-NAFL DNL(–) subjects (Figure 1H). The middle 2 quartiles (*n* = 11, Q2) had an average fasting DNL that was 60% higher than that of non-NAFL DNL(+) subjects, and the upper quartile (*n* = 6, Q3) had an average fasting DNL that was 90% higher than that of Q2 (Figure 1H). Although DNL generally correlated with hepatic TG, hepatic TG content did not differ significantly across the range of DNL (Q1–Q3) among NAFL subjects (Figure 1I). Likewise, plasma TG robustly correlated with fasting DNL, but quartiles of NAFL groups were not significantly different from each other (Figure 1J). Thus, persistent DNL was exacerbated in subjects with hepatic steatosis, but the degree of steatosis was not strongly predictive of the rate of fasting DNL.

### Fasting DNL is linked to lower ketogenesis and increased reliance on the TCA cycle.

**Figure 2:**
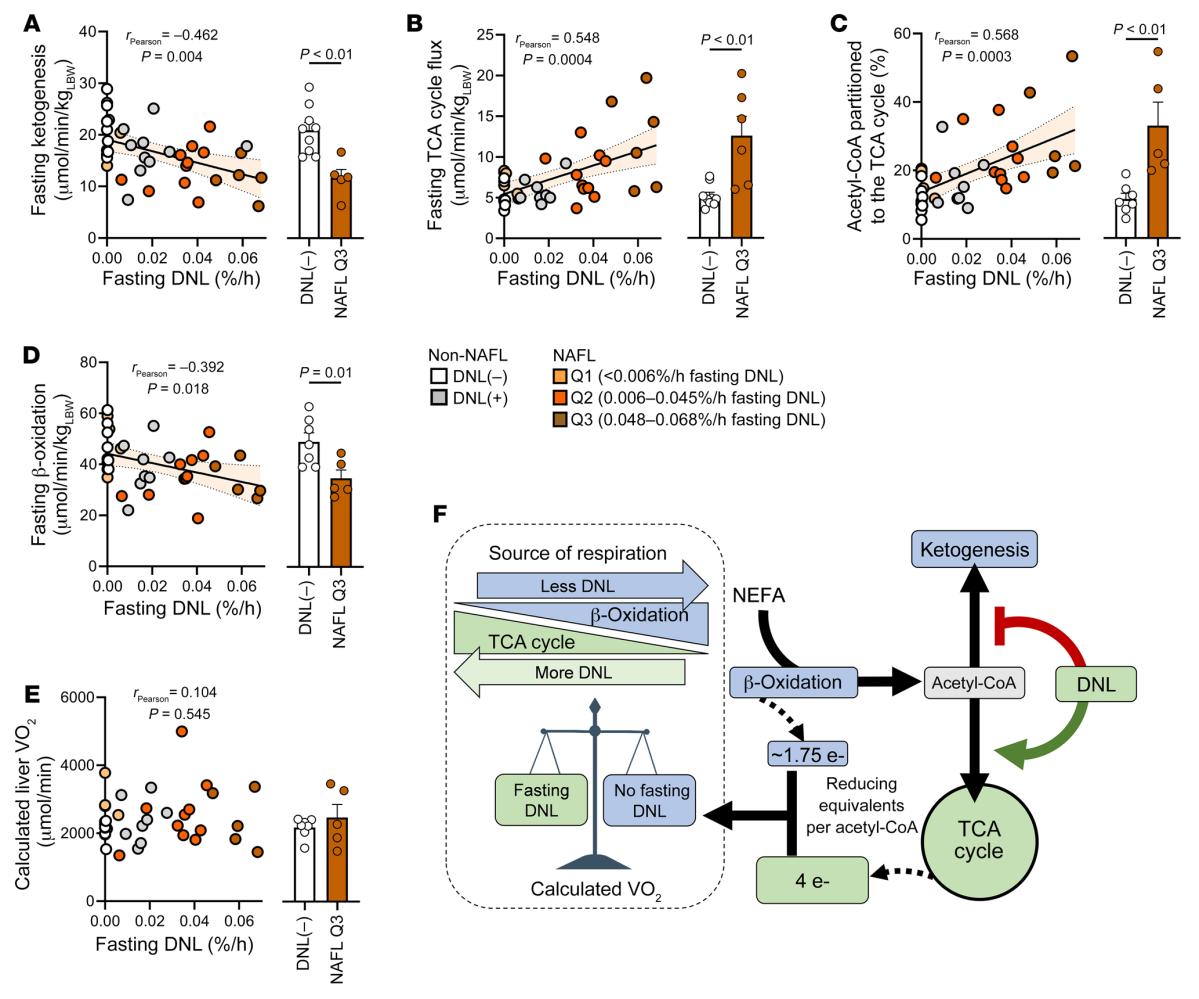
Fasting DNL in NAFL subjects is associated with lower ketogenesis and β-oxidation, but increased TCA cycle flux, which preserves estimated hepatic oxygen consumption. (**A**) Correlation between fasting DNL and ketogenesis measured by in vivo ketone turnover (*n* = 38), with comparison between subjects without NAFL (non-NAFL) and no DNL [DNL(–)] and NAFL subjects with the highest DNL (Q3). (**B**) Correlation between fasting DNL and TCA cycle flux measured by in vivo ^13^C and ^2^H isotopomer analysis of plasma glucose (*n* = 38), with comparison between DNL(–) and Q3 subjects. (**C**) Correlation between fasting DNL and the fraction of acetyl-CoA partitioned to the TCA cycle (vs. ketogenesis) (*n* = 36), with comparison between DNL(–) and Q3 subjects. (**D**) Inverse correlation between fasting DNL and the apparent rate of hepatic β-oxidation, determined as [TCA cycle flux] + [2 × ketogenesis] (*n* = 36), with a comparison between DNL(–) and Q3 subjects. (**E**) Oxygen consumption, calculated by flux balance analysis of net NADH/FADH_2_ production, did not correlate with fasting DNL (*n* = 36) and did not differ between DNL(–) and Q3 subjects. (**F**) Schema showing acetyl-CoA partitioning between ketogenesis and the TCA cycle during fasting DNL. The shift to TCA cycle metabolism preserves reducing equivalent flux and oxygen consumption when β-oxidation and ketogenesis are suppressed. e–, electron. Pearson correlations were used to test for statistically significant relationships. Statistical significance between groups was determined using a 1-way ANOVA (see Supplemental Figure 2 for all groups). Non-correlative data are presented as mean ± SEM.

Since the DNL intermediate malonyl-CoA negatively regulates mitochondrial fatty acid transport (Supplemental Figure 1) (18), we examined whether persistent fasting DNL was associated with altered hepatic oxidative metabolism. Ketogenesis inversely correlated with fasting DNL regardless of NAFL status, and NAFL subjects in the most severe DNL quartile (Q3) had a 2-fold lower rate of ketogenesis than non-NAFL DNL(–) control subjects (Figure 2A). In contrast, TCA cycle flux positively correlated with fasting DNL, including a greater than 2-fold increase in Q3 NAFL subjects (Figure 2B). Hence, the fraction of acetyl-CoA partitioned to the TCA cycle (versus ketogenesis) strongly correlated with the fractional rate of fasting DNL (Figure 2C). As a result, the apparent rate of hepatic β-oxidation (estimated as [TCA cycle flux] + [2 ∙ ketogenesis]) modestly but significantly correlated with fasting DNL and was significantly suppressed in Q3 NAFL subjects (Figure 2D). It has been suggested (3, 4) that the definition of normal liver TG should be lower than the 5% cutoff used here (1). When a 2% liver TG cutoff (3, 4) was applied to the cohort, the apparent rate of β-oxidation declined significantly across the NAFL quartiles of fasting DNL (Supplemental Figure 2). Thus, fasting DNL, especially in Q3 NAFL subjects, may limit β-oxidation and cause acetyl-CoA to be used in the TCA cycle rather than converted to ketones.

Despite the apparent decrease in hepatic β-oxidation, the higher reducing equivalent yield of the TCA cycle, compared with ketogenesis, preserved the estimated hepatic oxygen consumption (Figure 2E). Thus, decreased ketogenesis and increased reliance on the TCA cycle in NAFL subjects with fasting DNL–mediated inhibition of β-oxidation may be necessary to maintain normal ATP production and energy homeostasis in liver (Figure 2F).

### Fasting DNL reflects metabolic inflexibility.

**Figure 3:**
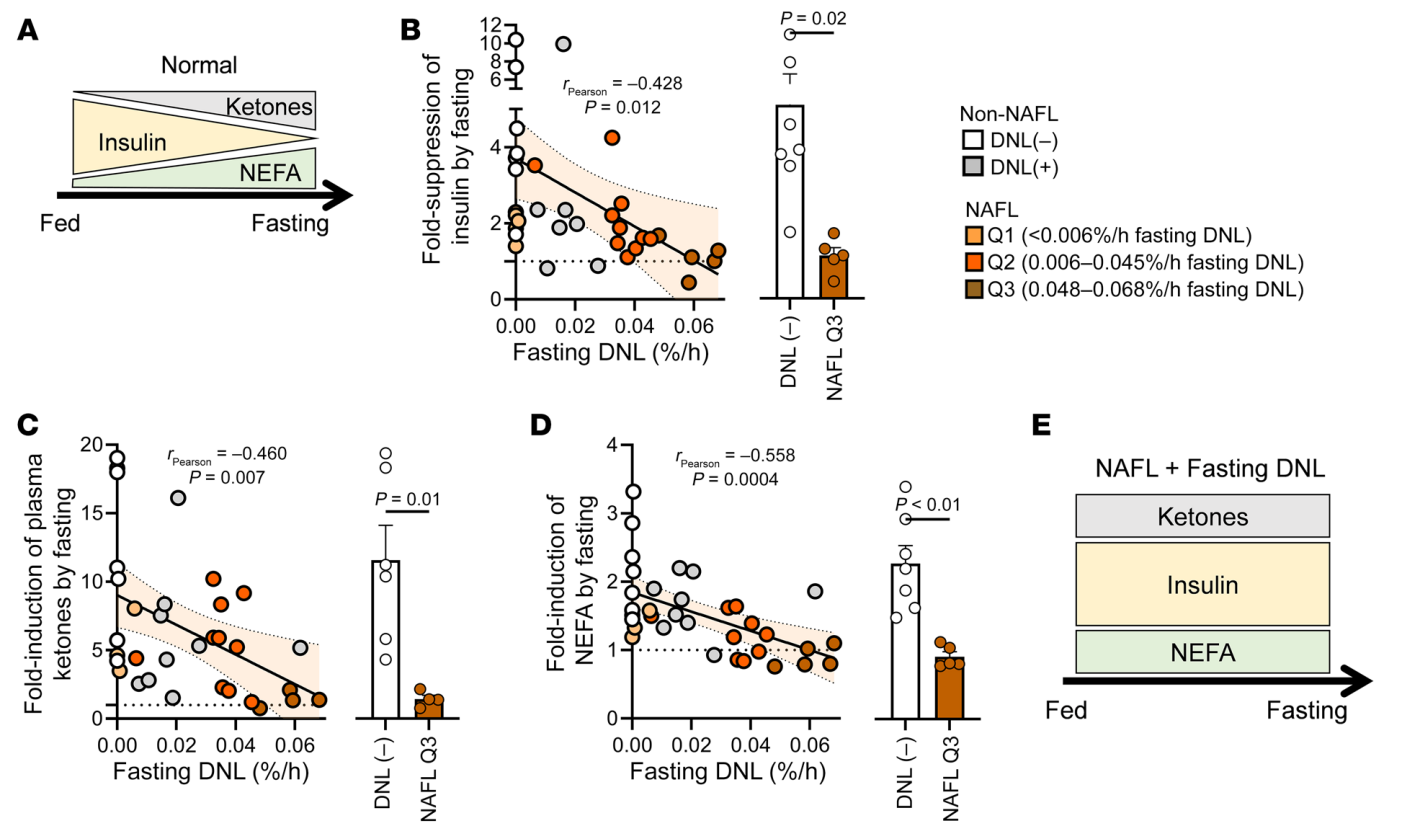
Subjects with persistent fasting DNL are intransigent to multiple effects of fasting. Fold changes were determined from plasma/serum measurements after an overnight and a 24-hour fast. (**A**) Expected physiological changes in normal humans transitioning from the fed to the fasting state. (**B**) Correlation between fasting DNL and the fold decrease in plasma insulin during a 24-hour fast (*n* = 34), with comparison between subjects without NAFL (Non-NAFL) and DNL [DNL(–)] and NAFL subjects with the highest DNL (Q3). (**C**) Correlation between fasting DNL and the fold increase in plasma ketones during a 24-hour fast (*n* = 34), with comparison between DNL(–) and Q3 subjects. (**D**) Correlation between fasting DNL and the fold increase in plasma non-esterified fatty acids (NEFAs) during a 24-hour fast (*n* = 36), with comparison between DNL(–) and Q3 subjects. (**E**) Persistent fasting DNL among NAFL subjects blunts the normal responses of insulin, ketones, and NEFA to fasting. Pearson correlations were used to test for statistically significant relationships. Statistical significance between groups was determined using a 1-way ANOVA (see Supplemental Figure 3 for all groups). Non-correlative data are presented as mean ± SEM.

Indirect calorimetry was consistent with adherence to the fasting protocol (21), but flux results suggested that metabolic characteristics of the fed state linger during fasting in some subjects. Therefore, plasma insulin, ketones, and NEFA were examined for their expected response to an overnight versus 24-hour fast (Figure 3A). Insulin, whose hepatic action stimulates DNL, fell 5-fold in non-NAFL DNL(–) subjects after a 24-hour fast. Remarkably, insulin suppression was lost with increasing fasting DNL and entirely failed to respond in NAFL Q3 subjects (Figure 3B). Non-NAFL DNL(–) subjects had a mean 12-fold increase in plasma ketones during a 24-hour fast, but this response declined across the spectrum of DNL regardless of NAFL status, and ketones failed to increase whatsoever in NAFL Q3 subjects (Figure 3C). Adipose lipolysis-derived plasma NEFA rose 2-fold in non-NAFL DNL(–) subjects, but the response declined with fasting DNL and was abolished entirely in those with the highest DNL (Figure 3D). Thus, persistent fasting DNL among NAFL subjects was associated with metabolic inflexibility in the broad response to fasting (Figure 3E). However, it is notable that the lack of responsiveness in NAFL subjects with fasting DNL was partially driven by basally elevated plasma insulin, ketones, and NEFA, which then failed to respond to the 24-hour fast (Supplemental Figure 3).

### The persistence of DNL during fasting is associated with insulin resistance.

**Figure 4:**
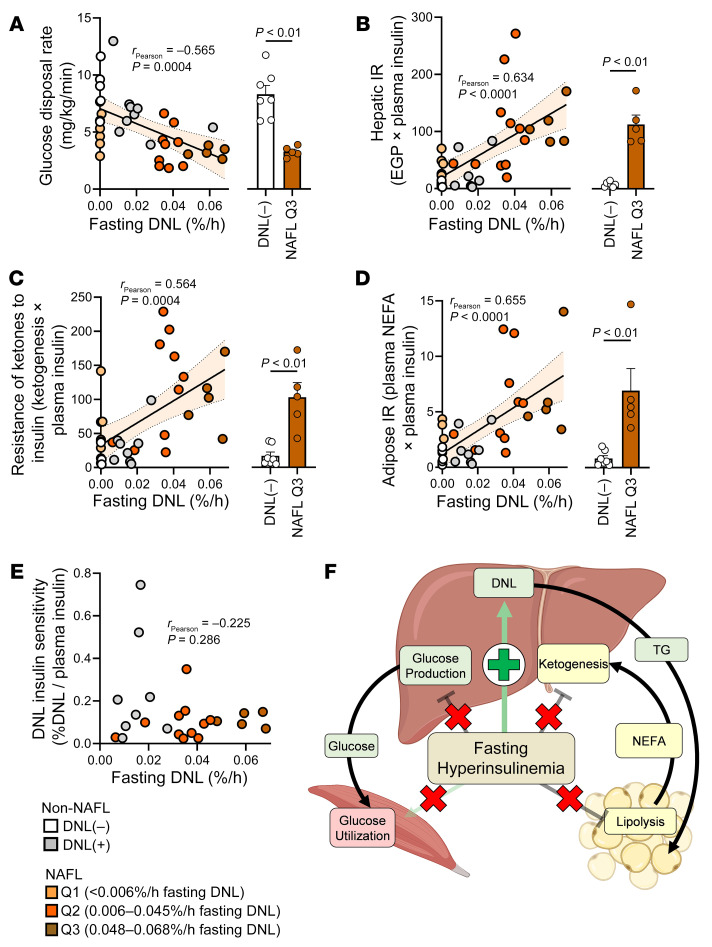
Persistent fasting DNL is associated with selective insulin resistance. (**A**) Correlation between fasting DNL and glucose disposal during a hyperinsulinemic-euglycemic clamp (*n* = 35). (**B**) Correlation between fasting DNL and hepatic insulin resistance (*n* = 35), measured as endogenous glucose production (EGP) indexed to plasma insulin. (**C**) Correlation between fasting DNL and ketogenic insulin resistance (*n* = 35), measured as endogenous ketone production indexed to plasma insulin. (**D**) Correlation between fasting DNL and adipose insulin resistance (IR) (*n* = 35), measured as plasma NEFA indexed to plasma insulin. (**E**) The sensitivity of DNL to insulin is represented as DNL relative to plasma insulin (*n* = 36). (**F**) Persistent fasting DNL in NAFL subjects is associated with hepatic insulin resistance in glucose and ketone production pathways but not DNL, which retains responsiveness to hyperinsulinemia. Pearson correlations were used to test for statistically significant relationships. Statistical significance between groups was determined using a 1-way ANOVA (see Supplemental Figure 4 for all groups). Non-correlative data are presented as mean ± SEM.

Insulin normally suppresses endogenous glucose production (EGP), adipose lipolysis, and ketogenesis, but it activates DNL and peripheral glucose disposal (Supplemental Figure 4A). Plasma glucose and EGP were elevated in NAFL subjects with the highest fasting DNL (Supplemental Figure 4, B and C). Peripheral insulin sensitivity, indicated by glucose disposal during a hyperinsulinemic-euglycemic clamp (Figure 4A) and hepatic insulin resistance, indexed as EGP × [insulin] (Figure 4B), worsened with increased fasting DNL. Despite lower ketogenesis in NAFL subjects with persistent fasting DNL, ketogenesis was disproportionately high relative to insulin (ketone production × [insulin]), consistent with insulin resistance (Figure 4C). Likewise, adipose insulin resistance, indexed as plasma [NEFA] × [insulin], was increased in all NAFL groups (Figure 4D). In contrast, when DNL was normalized to plasma insulin, there were no differences between groups where DNL was detected [i.e., DNL(+), Q2, or Q3] (Figure 4E). Thus, DNL in NAFL subjects was related to profound but selective hepatic insulin resistance in glucose and ketone production pathways, but not DNL, which retained responsiveness to hyperinsulinemia (Figure 4F), as previously described (5).

### Oxaloacetate is shifted from gluconeogenesis to citrate synthesis in subjects with elevated fasting DNL.

**Figure 5:**
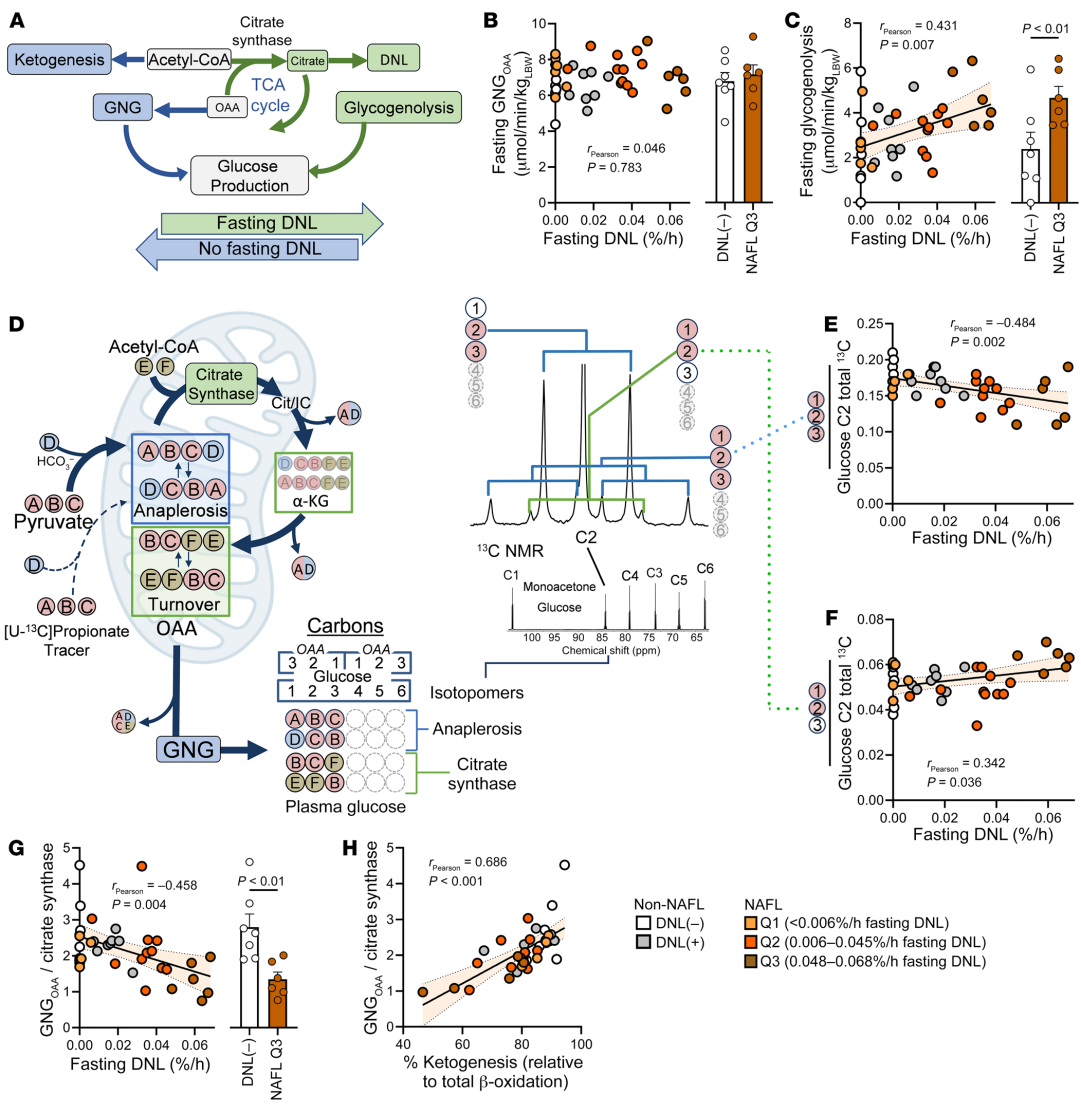
Hepatic oxaloacetate is preferentially utilized for citrate synthesis in subjects with persistent fasting DNL. (**A**) The competition for oxaloacetate (OAA) by gluconeogenesis (GNG) and citrate synthase impacts the fate of acetyl-CoA. Abundant oxaloacetate facilitates citrate synthase, which outcompetes ketogenesis for acetyl-CoA. The resulting citrate is either oxidized back to oxaloacetate in the TCA cycle or transported from the mitochondria for DNL. (**B** and **C**) The absolute rate of gluconeogenesis from oxaloacetate (*n* = 38) (**B**) and glycogenolysis (*n* = 38) (**C**) was measured by glucose turnover and ^2^H incorporation into positions C2, C5, and C6 of plasma glucose. (**D**) A [U-^13^C_3_]propionate tracer was used to track oxaloacetate’s relative fate between gluconeogenesis and citrate synthesis. Anaplerosis was traced as [1,2,3-^13^C] or [2,3,4-^13^C] oxaloacetate isotopomers, which were diluted to [1,2-^13^C] or [3,4-^13^C] isotopomers upon reaction with unlabeled acetyl-CoA in the citrate synthase reaction and a subsequent turn of the TCA cycle (13). These atoms were transmitted to plasma glucose by gluconeogenesis, and isotopomers were detected by ^13^C-NMR and analysis of the C2 multiplets. (**E**) Negative correlation between fasting DNL and isotopomers arising from anaplerosis and gluconeogenesis (*n* = 38). (**F**) Positive correlation between fasting DNL and isotopomers arising from citrate synthase (*n* = 38). (**G**) Negative correlation between fasting DNL and the utilization of oxaloacetate for gluconeogenesis relative to its use by citrate synthase (*n* = 38). (**H**) Positive correlation between acetyl-CoA used for ketogenesis and the utilization of oxaloacetate for gluconeogenesis relative to its use by citrate synthase (*n* = 38). Pearson correlations were used to test for statistically significant relationships. Statistical significance between groups was determined using a 1-way ANOVA. Non-correlative data are presented as mean ± SEM.

A shift in the fate of acetyl-CoA from the TCA cycle to ketogenesis occurs when hepatic oxaloacetate is depleted by gluconeogenesis and other factors during fasting (27), which limits citrate synthase flux and promotes the partitioning of acetyl-CoA to ketones (34, 35) (Figure 5A). Since ketogenesis, gluconeogenesis, and DNL are influenced by oxaloacetate availability in the TCA cycle, we examined this network as a potential link between increased fasting DNL and decreased ketogenesis. We previously reported that this cohort of NAFL subjects had increased glucose production and gluconeogenesis (21). However, deuterium incorporation into the C2, C5, and C6 positions of glucose indicated that the fraction of glucose production originating from gluconeogenesis decreased, and the fraction of glucose production originating from glycogenolysis increased, with fasting DNL (Supplemental Figure 5, A–C). The positive effect of NAFL on absolute rates of gluconeogenesis from oxaloacetate (GNG_OAA_) (21) was preserved in the Q2 DNL group. However, this effect was lost in the Q3 subgroup of NAFL subjects with the highest fasting DNL (Figure 5B), who maintained elevated EGP (Supplemental Figure 4C) by increased rates of glycogenolysis (Figures 5C).

The increased availability of glycogenolysis may spare hepatic oxaloacetate. Unfortunately, this TCA cycle intermediate cannot be noninvasively measured. However, the fluxes that contribute to its formation and utilization can be assessed by tracer approaches (13). Oxaloacetate consumed by the citrate synthase reaction is regenerated every turn of the TCA cycle, but its availability for citrate synthesis is influenced positively by anaplerosis and negatively by its cataplerotic loss to gluconeogenesis (13). These relative activities were measured by ^13^C-NMR isotopomer analysis of plasma glucose, which partially originates from oxaloacetate during gluconeogenesis (Figure 5D). The isotopomers of glucose that indicate gluconeogenesis negatively correlated with fasting DNL (Figure 5E), while the isotopomers that indicate citrate synthase activity were positively associated with fasting DNL (Figure 5F). These data were used to determine relative rates (36). There was a modest but significant positive correlation between DNL and the absolute rates of anaplerosis and pyruvate cycling (Supplemental Figure 5, D and E). However, there was a robust negative correlation between DNL and the rate of oxaloacetate lost to gluconeogenesis relative to its utilization for citrate synthesis (GNG_OAA_/citrate synthase) (Figure 5G). The loss of oxaloacetate to gluconeogenesis relative to its conversion to citrate also correlated with the partitioning of acetyl-CoA to ketogenesis (Figure 5H). In other words, increased utilization of oxaloacetate for citrate synthesis may partially reflect the reliance of DNL on TCA cycle intermediates (e.g., citrate) for the citrate shuttle (13, 37) and simultaneously diminish ketogenesis (Figure 5A).

### Increased fasting DNL is associated with elevated liver size and perturbed liver function.

**Figure 6:**
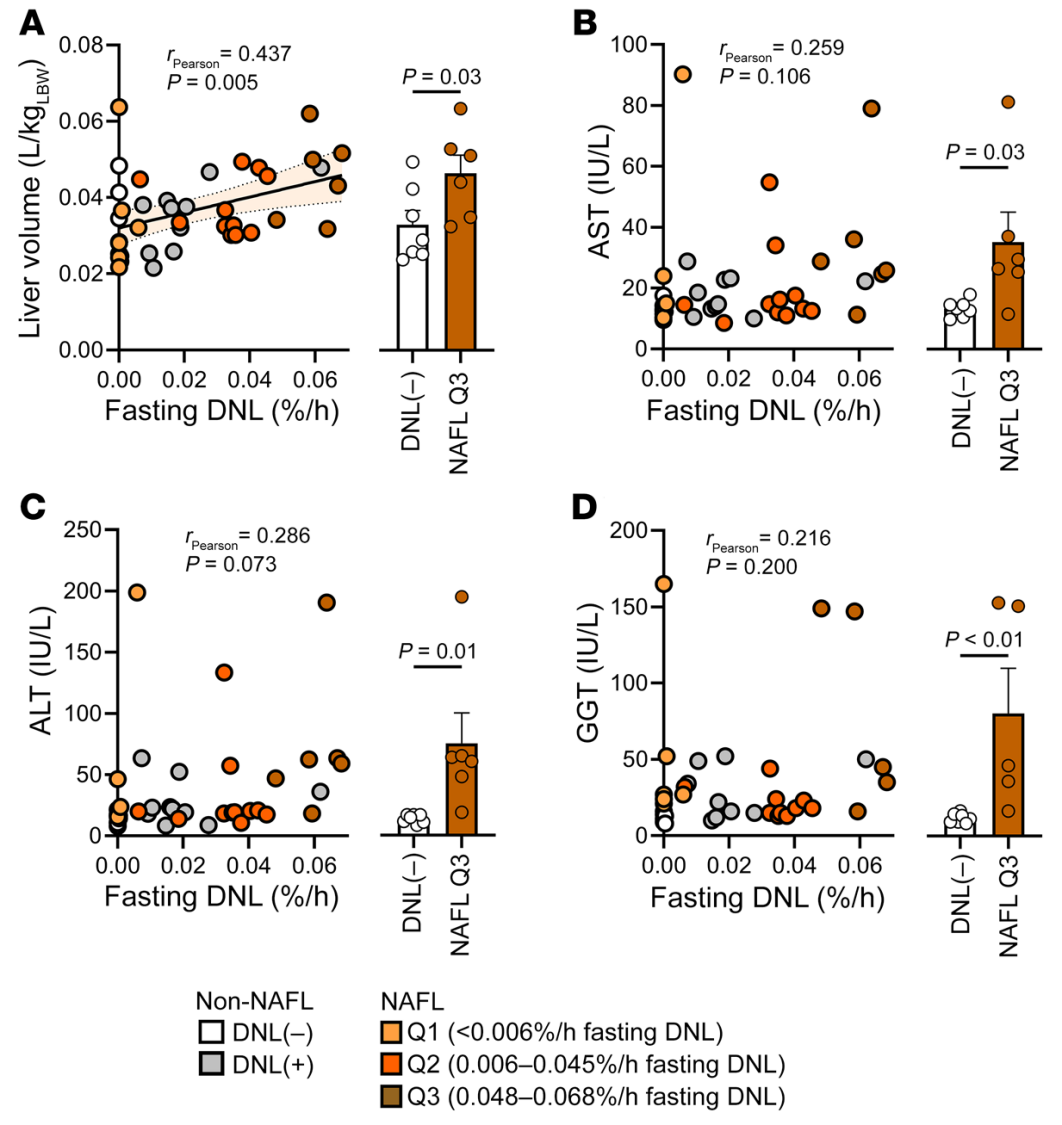
Elevated liver volume, but not other indices of liver function, correlates with fasting DNL. (**A**) Hepatic volume positively correlated with fasting DNL activity (*n* = 40). (**B**–**D**) Relationship between fasting DNL and plasma aspartate aminotransferase (AST) (**B**), alanine aminotransferase (ALT) (**C**), and γ-glutamyltransferase (GGT) (**D**) (*n* = 40 per panel). Pearson correlations were used to test for statistically significant relationships. Statistical significance between groups was determined using a 1-way ANOVA (see Supplemental Figure 6 for all groups). Non-correlative data are presented as mean ± SEM.

Since individuals with persistent fasting DNL had marked insulin resistance and altered metabolic flux, but a wide range of steatosis, we examined whether persistent fasting DNL was related to clinical indicators of liver dysfunction. Factors that induce DNL during insulin resistance, such as hyperinsulinemia and mTORC1 signaling, may also stimulate growth pathways, suppress autophagy, and promote hepatomegaly (38, 39), which predicts more severe liver disease (40, 41). Hepatic volume estimated by cross-sectional imaging positively correlated with fasting DNL and was significantly greater among NAFL subjects with the highest fasting DNL (Q3) compared with non-NAFL DNL(–) subjects (Figure 6A). However, liver function indices, including aspartate aminotransferase, alanine aminotransferase, and γ-glutamyl transferase (Figure 6, B–D), correlated poorly with fasting DNL but were higher in the Q3 NAFL group than in the non-NAFL DNL(–) group. These indices were also higher in non-NAFL DNL(+) subjects than in their DNL(–) counterparts, though not to a clinically significant degree (Supplemental Figure 6). Thus, more detailed studies are required to determine whether persistent fasting DNL and related factors affect liver health.

## Discussion

Lipogenesis remained detectable in a subset of humans after a 24-hour fast, including some subjects without hepatic steatosis or other hallmarks of metabolic syndrome. Lipogenic individuals displayed reduced ketogenesis but increased acetyl-CoA utilization in the TCA cycle. Subjects with NAFL in the highest quartile of fasting DNL had the lowest rates of β-oxidation, consistent with the canonical role of malonyl-CoA in regulating mitochondrial NEFA transport (17). At least 3 metabolic factors distinguished those who manifested persistent DNL during fasting. Firstly, they were resistant to the typical physiological effects of fasting, such as attenuation of fasting plasma insulin concentrations, augmentation of fasting plasma NEFA and ketone concentrations, and a diminution of glycogenolysis. Secondly, and concordantly, persistent lipogenesis in these subjects appeared to be a proportionate response to their relative hyperinsulinemia, despite the inability of hyperinsulinemia to suppress hepatic glucose production. Thirdly, subjects with persistent fasting DNL had lower rates of oxaloacetate utilization for gluconeogenesis compared with its utilization for citrate synthesis, which may inhibit ketogenesis. Thus, despite being only a fraction of the rate of postabsorptive DNL, 24-hour fasted DNL is associated with marked disruptions in lipid oxidation.

The inability to suppress DNL during fasting distinguished subgroups of non-NAFL and NAFL subjects. Subjects who did not have hepatic steatosis but had persistent lipogenesis after a 24-hour fast tended to resemble NAFL subjects without measurable fasting DNL activity (Q1), including having higher liver function indices compared with non-NAFL DNL(–) subjects. Likewise, NAFL subjects in DNL quartile Q1 had a liver TG content that was not significantly different from that of Q2 and Q3 but had lower hepatic insulin resistance and relatively normal metabolism through pathways of ketogenesis, the TCA cycle, and gluconeogenesis. Conversely, subjects with NAFL and the highest levels of DNL (Q3) also had the highest TCA cycle flux and lowest rates of ketogenesis and were the only group with significantly reduced rates of β-oxidation. Although liver function indices and hepatic insulin resistance of NAFL DNL quartile Q3 were not distinguishable from those of Q1 and Q2, this group had the largest liver volume, which may suggest an increased risk for liver disease (40, 41). Notably, these comparisons are based on a 5% liver TG threshold for NAFL, but if 2% is used as recently suggested (3, 4), then the distinctions between the groups are retained or amplified. Nevertheless, strong correlations existed between fasting DNL, ketogenesis, and TCA cycle metabolism regardless of the NAFL threshold.

The parallel activation of TCA cycle metabolism and DNL is consistent with the role of the citrate shuttle in the export of mitochondrial acetyl-CoA for cytosolic lipogenesis (13, 37) (Supplemental Figure 1). Maintaining DNL requires increased TCA cycle intermediates for citrate formation, thereby attenuating ketogenesis via substrate competition for acetyl-CoA. Previous studies in overnight-fasted individuals, in whom ketogenesis is minimal, also found increased TCA cycle flux in NAFL subjects (10). A parallel study of the same subjects revealed a 4-fold higher rate of DNL (6), suggesting that elevated DNL is closely associated with the activation of TCA cycle metabolism in NAFL subjects, regardless of ketogenic state. Importantly, the hormonal and/or substrate conditions that drive excess DNL in NAFL subjects also impinge on pathways related to mitochondrial function. Prior ex vivo studies indicate that hepatic mitochondrial respiration is either normal or compensated to higher oxygen consumption in NAFL subjects (12, 14) and eventually declines with advanced disease and the onset of steatohepatitis and/or diabetes (16, 42). However, there is general agreement that the mitochondria of NAFL subjects are prone to ROS production, which is thought to contribute to hepatocellular damage (2). Since DNL requires TCA cycle metabolism and limits ketogenesis, the inability to suppress DNL may have secondary and deleterious consequences for apparent mitochondrial function in NAFL, even without morphological defects or ex vivo respiratory capacity.

Selective hepatic insulin resistance impairs the ability of insulin to suppress glucose production but not its ability to activate lipogenesis (5). Subjects with NAFL and persistent fasting DNL had more severe hepatic insulin resistance with respect to endogenous glucose production compared with both non-NAFL and NAFL subjects without fasting DNL. This finding is consistent with a recent study that found a strong correlation between insulin resistance and the aberrant activation of DNL in NAFL subjects (9). Likewise, insulin resistance indexed to ketogenesis (i.e., ketogenesis × insulin) was generally correlated with DNL. However, this index was not significantly different among NAFL groups with or without DNL, which may contribute to lower fasting ketones in subjects with higher insulin. Likewise, DNL normalized to insulin was not different in any groups in which DNL was detected. In rodent models, the activation of DNL during insulin resistance can be attributed to sustained mTORC1 signaling (38). In mice with constitutively active mTORC1, examined using similar in vivo tracer techniques, TCA cycle flux was sustained, and ketogenesis was suppressed during fasting (39). Notably, NAFL subjects with the most profound fasting DNL (Q3) also had increased liver size, an effect observed in mice with constitutive mTORC1 signaling (39). Thus, hyperinsulinemia may promote DNL and liver size in some NAFL subjects.

It was recently reported that increased DNL in NAFL subjects results from increased substrate availability (43), which may also contribute to the disruption of several intrinsic metabolic mechanisms that usually promote ketogenesis and inhibit the TCA cycle during fasting. First, gluconeogenic demand for cataplerosis is expected to outpace anaplerotic substrate availability during fasting, resulting in a decline in oxaloacetate, the inhibition of citrate synthase, and the activation of ketogenesis (26, 27). In subjects with elevated fasting DNL, ^13^C-NMR isotopomer analysis of plasma glucose indicated that the relative bifurcation of oxaloacetate between gluconeogenesis and citrate synthesis was shifted toward the latter. This shift may indicate sustained oxaloacetate availability during fasting, consistent with higher rates of glycogenolysis, lower ketogenesis, and increased TCA cycle flux. Elevated glycogenolysis is likely an indicator of increased substrate availability, inasmuch as glycogen depletion is a necessary factor for the induction of ketogenesis (24). Second, citrate synthesis sustained by increased substrate availability may facilitate mitochondrial citrate export to supply cytosolic acetyl-CoA and the fatty acid synthase precursor malonyl-CoA, which also inhibits β-oxidation (17). The inverse correlation between β-oxidation and DNL and, more specifically, reduced β-oxidation in NAFL subjects with the highest DNL are consistent with this mechanism.

Alternatively, the activation of DNL during fasting can also result from impaired function of the ketogenic pathway. Early investigations indicated that the fate of acetyl-CoA is dictated by a competition between ketogenesis and lipid synthesis rather than oxidation in the TCA cycle (24). Accordingly, inhibition of ketogenesis in mice caused increases in citrate synthase flux, glycogenolysis, and lipid synthesis (44). The increased reliance on the TCA cycle in those mice, as in NAFL subjects, is likely required to maintain cellular energy charge. Despite lower β-oxidation in NAFL subjects with the highest DNL, hepatic oxygen consumption estimated from measured fluxes was not decreased. Oxidative phosphorylation was putatively sustained by the increased yield of reducing equivalents from the TCA cycle compared with β-oxidation alone. In the context of ketogenic insufficiency, acetyl-CoA generation exceeding cellular energy demand was shunted to lipids following citrate formation rather than conversion to ketones or complete oxidation (44). Notably, ketogenic insufficiency results in more significant hepatic injury in mice (45), perhaps due to the preferential synthesis of cytotoxic lipid species (44). The data from this human study cannot distinguish these various mechanisms.

Finally, a recent study of NASH subjects used multiple ^2^H_2_O dosing periods and plasma TG-palmitate enrichment measurements over 12 weeks to model lipid turnover (46). Data from NASH subjects indicated 2 pools of lipid, one pool that turns over rapidly (half-life ~3.4 hours) and one that turns over very slowly (half-life ~13 days) (46). If TG pool dynamics vary between normal and NAFL subjects, then the single 15-hour measurement of TG-palmitate enrichment used here may underestimate DNL in subjects with pools of TG that turn over more slowly, as described in previous simulations of hepatic TG dynamics (47). To theoretically examine the effect of TG pool size on estimates of fractional DNL (percentage per hour), we modeled palmitate enrichment kinetics over 80 hours using the hepatic TG content (estimated from ^1^H magnetic resonance spectroscopy data), disposal rates (minimally estimated as 50% β-oxidation and 50% TG secretion), and the palmitate enrichment at 15 hours (Supplemental Figure 7A). A single-pool model of TG turnover predicted half-lives of approximately 3 hours in non-NAFL subjects, which increased more than 20-fold in NAFL subjects, primarily because of their larger pool size (Supplemental Figure 7B). Thus, it is notable that the fractional DNL reported here may be underestimated for fasting NAFL subjects relative to non-NAFL subjects. However, the general trend was similar to the fractional rates (Supplemental Figure 7C). Nevertheless, these absolute flux estimates remain theoretical, since extended temporal sampling is not feasible during fasting.

In conclusion, elevated DNL and hepatic TCA cycle function are metabolically linked in 24-hour-fasted subjects. The simultaneous preservation of TCA cycle metabolism and DNL but suppression of ketogenesis in some NAFL subjects appears to be the consequence of reciprocal regulation of DNL and β-oxidation. Additional studies are required to determine whether the interplay between mitochondrial metabolism and DNL in NAFL is mediated by substrate, redox, and/or signaling factors and whether the inability to suppress fasting DNL is a mechanistic event in the onset and progression of NAFL.

## Methods

### Participants.

Forty consecutive nondiabetic individuals with and without a history of NAFL were recruited for this observational study at the University of Texas Southwestern (UTSW) Medical Center between 2011 and 2016. The Hepatology practice at UTSW served as the primary source of recruitment for subjects with NAFL. Additional NAFL subjects, as well as non-NAFL volunteers, were recruited from the UTSW campus via flyers and media. Subjects consuming alcohol in excess (>30 g/d in men, >20 g/d in women), with a BMI greater than 35 kg/m^2^, with concurrent liver disease (other than NAFL), taking medications associated with hepatic steatosis, or participating in a regular exercise program were excluded from the study. A total of 67 subjects were recruited, of whom 27 were dropped from the study due to screening failure (*n* = 9), scheduling issues (*n* = 11), or problems encountered with i.v. access during the isotope infusion protocol (*n* = 7). Data from these subjects were previously collated according to liver steatosis (21). The study was originally powered to detect a minimum difference in ketogenesis of 4.6 μmol/min/kg_Lean Body Weight_ (β = 0.80, α = 0.05).

### Study design.

The study design was previously described for these subjects (21). Briefly, 1 week before isotope studies, all subjects underwent dual-energy x-ray absorptiometry (DEXA) to assess body fat distribution and lean mass, ^1^H magnetic resonance spectroscopy (^1^H-MRS) to determine hepatic TG content, and hyperinsulinemic-euglycemic (H-E) clamp to measure insulin sensitivity (Figure 1A). Subjects then maintained an ad libitum diet for 4 days, keeping a record of food intake to allow the individual daily caloric intake to be determined. Subjects were then placed on a 3-day standard meal plan prepared by the Clinical and Translational Research Center kitchen, consisting of 50% carbohydrate, 35% fat, and 15% protein with a daily caloric intake reflective of their usual diet. The final standard meal occurred at 12:00 pm the day before isotopic studies, marking the beginning of a 24-hour fast. Subjects presented to the Advanced Imaging Research Center (AIRC) at 8:00 am the following day to undergo the isotope studies to assess in vivo hepatic metabolism. Indirect calorimetry was performed before the H-E clamp and the isotope study (Vmax Encore, CareFusion). All subjects underwent the same protocol, and the cross-sectional design obviated the need for randomization or blinding.

### Hyperinsulinemic-euglycemic clamp.

The H-E clamp in these subjects was previously described (21). Briefly, 1 week before the isotopic studies and after an overnight fast (last meal at 6:30 pm), subjects underwent an H-E clamp. The H-E clamp could not be completed in 1 control and 3 NAFL subjects.

### Dual-energy x-ray absorptiometry.

Fat mass, fat-free mass, and bone mineral mass in the total body, trunk, lower body, and upper extremities were measured using DEXA scanning as previously described (48).

### Hepatic TG content.

Measurement of hepatic TG in these subjects was previously described (21). Those with a hepatic TG content of less than 5% were considered non-NAFL subjects (*n* = 17), and the remainder were considered to be NAFL subjects (*n* = 23). Liver volume (*V*_liver_) was determined from cross-sectional images obtained during ^1^H-MRS (49). Briefly, the largest craniocaudal (cc), ventrodorsal (vd), and coronal (cor) diameters were measured, and the following equation was used to estimate liver volume: *V*_liver_ = cc × vd × cor × 0.31.

### Isotopes and other materials.

Seventy percent [^2^H]water and 99% [U-^13^C_3_]propionate (sodium salt) were obtained from Cambridge Isotopes. Sterility- and pyrogen-tested [3,4-^13^C_2_]glucose (98%) was obtained from Omicron Biochemicals Inc. in sealed, stoppered vials. Sterility- and pyrogen-tested [3,4-^13^C_2_]ethylacetoacetate (98%) and [1,2-^13^C_2_]sodium β-hydroxybutyrate (98%) were obtained from Isotec in sealed, stoppered vials. Other common reagents were purchased from MilliporeSigma.

### Isotope infusion protocol.

Tracer administration in these subjects was previously described (21). Briefly, between 10:00 pm the day before the study and 12:00 pm the day of the study, subjects received 2 tracers orally: divided doses of 70% [^2^H]water (5 g/kg body water, calculated as 60% of body weight in men and 50% of body weight in women) at 10:00 pm, 9:00 am, and 10:30 am and [U-^13^C_3_]propionate (~400 mg) at 11:00 am, 11:30 am, and 12:00 pm. Subjects were provided with 0.5% [^2^H]water ad libitum for the remainder of the study. Infusions of [3,4-^13^C_2_]glucose, [3,4-^13^C_2_]acetoacetate, and [1,2-^13^C_2_]β-hydroxybutyrate began at 24-hour fasting and concluded 2 hours later with a 50 cc blood draw.

### Metabolic analysis.

Analyses of glucose turnover, gluconeogenesis, glycogenolysis, ketone turnover, and TCA cycle fluxes and flux balance analysis of hepatic oxygen consumption in these subjects were previously described (21). Glucose and TCA cycle analysis failed in 2 of 17 non-NAFL subjects. Ketogenesis analysis failed in 2 of 23 NAFL subjects. Thus, β-oxidation and estimated hepatic oxygen consumption were evaluated in 15 of 17 non-NAFL subjects and 21 of 23 NAFL subjects.

### DNL measurement by high-resolution Orbitrap gas chromatography–mass spectrometry.

Samples were collected after a 24-hour fast, but ^2^H_2_O was administered 9 hours into the fast (i.e., 15 hours before the collection). The body water enrichment was measured in plasma by analysis of acetone after exchange under basic conditions (50, 51). We have previously shown that low ^2^H enrichment of water can be analyzed by use of high-resolution Orbitrap gas chromatography–mass spectrometry in negative chemical ionization mode with targeted selected ion monitoring acquisition (*m/z* 55.5–60.5) and 60,000 mass resolution (full width half maximum, *m/z* 200). The sensitivity of the method is well suited for human studies given that deuterium labeling of body water is typically low (0.2%–0.5%) owing to side effects encountered with excessive loading (vertigo, nausea, etc.).

Lipids were isolated as previously reported (33). In brief, proteins were precipitated by addition of 2 mL (1:1 vol/vol) methanol/dichloromethane (DCM) in 5 μL of plasma. Samples were then vortexed and centrifuged for 5 minutes at 2,500 *g*. Next, the supernatant was transferred to a new tube and dried under N_2_. Dried extracts were then saponified with 1 mL 0.5 M KOH in methanol at 80°C for 1 hour; samples were cooled to room temperature and extracted with DCM/water. After the samples were vortexed, they were centrifuged for 5 minutes, and the bottom organic layer was separated. Next, the dried lipid extract was resuspended in 50 μL of 1% triethylamine in acetone, and derivatized with 50 μL of 1% pentafluorobenzyl bromide in acetone. After 30 minutes at room temperature, 1 mL iso-octane was added, and 1 μL was injected for mass spectrometric analysis.

The calculation of DNL was performed as previously described (33) and was based on the formula reported by Beylot and colleagues (52). Briefly, DNL is measured based on the incorporation of ^2^H into TG-palmitate following the oral administration of ^2^H_2_O. The contribution of palmitate synthesis was determined using the following equation: fractional DNL (%) = (palmitate ^2^H enrichment) / (water ^2^H enrichment × *n*) × 100, where DNL is the fraction of palmitate that was newly synthesized since the administration of deuterium oxide, palmitate ^2^H enrichment is expressed as described above, water ^2^H enrichment is the fraction of ^2^H_2_O in plasma, and *n* represents the number of exchangeable hydrogens on palmitate, which was measured to be 22 in our setup (33) and is similar to other reports. Since the fraction of new palmitate is also dependent on the duration of exposure, the fractional enrichment was divided by the duration of ^2^H_2_O exposure (percent per hour). This expression was used to compare DNL in 24-hour-fasted subjects who had ^2^H_2_O for 15 hours versus DNL in overnight-fasted subjects who had ^2^H_2_O for 2 hours (Figure 1A). A fractional DNL of 0.10% was considered to be the lower limit of detection and was used as a cutoff to demarcate those with and without detectable DNL after fasting (33).

### Statistics.

Statistical analyses were performed using Prism 9.0 (GraphPad Software LLC). Differences between groups were evaluated using the 2-tailed Student’s *t* test for paired and unpaired data and ANOVA as appropriate. The strength and significance of correlations were determined by Pearson analysis. Values are presented as mean with SEM unless otherwise indicated. Statistical significance was taken at *P* less than 0.05.

### Study approval.

This study was conducted according to Declaration of Helsinki principles and approved by the Institutional Review Board at UTSW. Each participant provided written informed consent before participation. ClinicalTrials.gov identifier: NCT02690792.

## Author contributions

JDB and SCB were responsible for conceptualization, data curation, formal analysis, funding acquisition, investigation, methodology, project administration, resources, supervision, visualization, writing of the original draft, and review and editing of the manuscript. XF and JAF were equally responsible for data curation, formal analysis, investigation, methodology, writing of the original draft, and review and editing of the manuscript. SD was responsible for review and editing of the manuscript, data curation, formal analysis, and investigation. MIV was responsible for data curation, formal analysis, and investigation.

## Supplementary Material

Supplemental data

Trial reporting checklists

ICMJE disclosure forms

## Figures and Tables

**Table 1:**
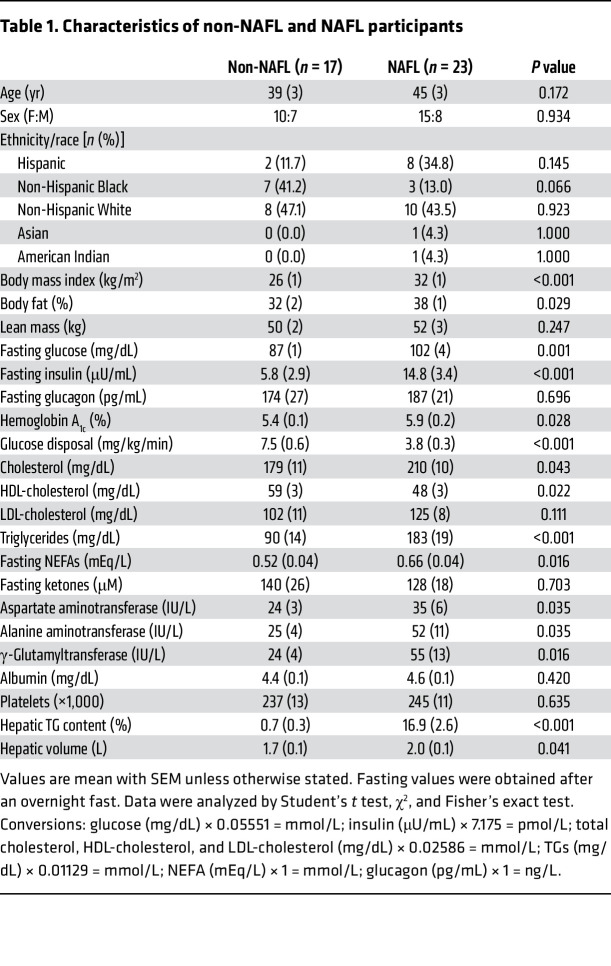
Characteristics of non-NAFL and NAFL participants

**Table 2:**
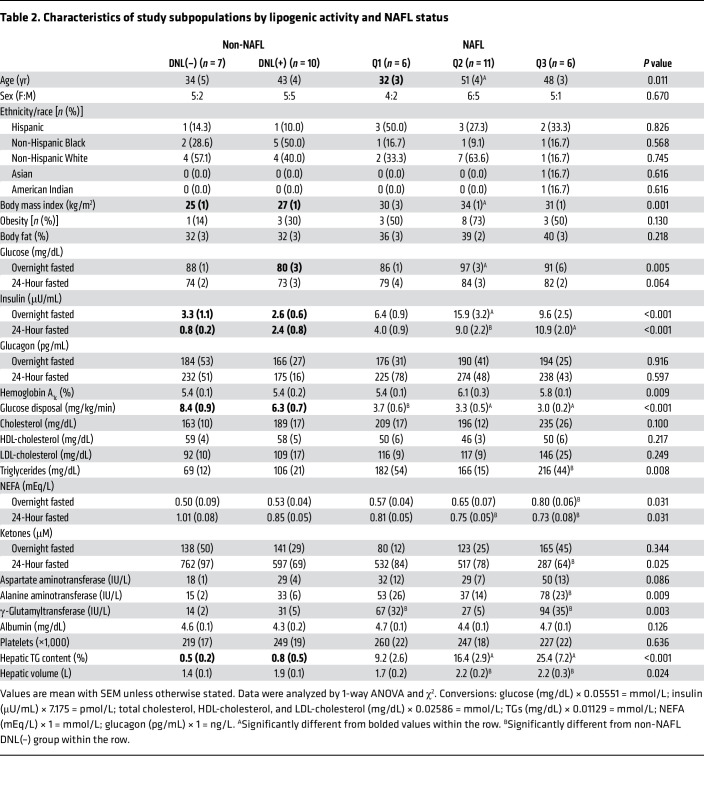
Characteristics of study subpopulations by lipogenic activity and NAFL status

## Version 1 03/16/2023

In-Press Preview

## Version 2 05/01/2023

Electronic publication
