# Supplementary material for: Persistent fasting lipogenesis links impaired ketogenesis with citrate synthesis in humans with nonalcoholic fatty liver
Source: J Clin Invest. 2023 May 1;133(9):e167442. doi: 10.1172/JCI167442 (PMC10145942; doi:10.1172/JCI167442)
Supplement: Supplemental data [file jci-133-167442-s030.pdf]

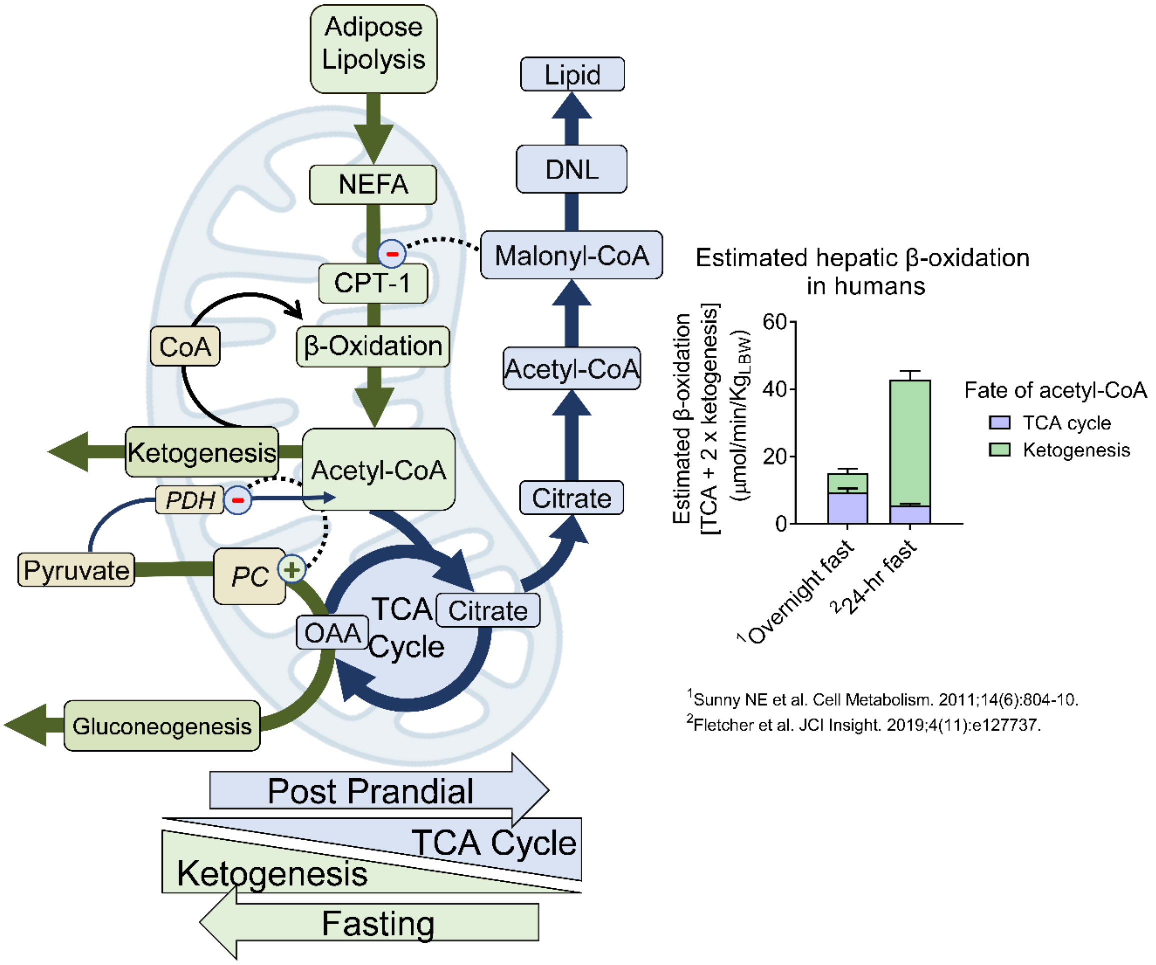

Supplemental Figure S1. Summary of metabolic pathways discussed in this study and some key allosteric regulators. Adipose lipolysis leads to the delivery of circulating non-esterified fatty acids (NEFA) to liver followed by transport into mitochondria by carnitine acyltransferase (CPT-1) and subsequent β-oxidation to yield acetyl-CoA and NADH/$FADH_2$. Acetyl-CoA can be converted to ketones or enter the tricarboxylic acid (TCA) cycle via condensation with oxaloacetate (OAA) to form citrate. Citrate can either be oxidized within the TCA cycle to yield CO_2 and NADH/$FADH_2$ or be exported to the cytosol via the citrate transporter, where it is converted back to acetyl-CoA and participates in de novo lipogenesis (DNL). Notably, the first committed step of lipogenesis is the formation of malonyl-CoA, which acts as a negative regulator of CPT-1, thereby limiting NEFA entry into mitochondria and β-oxidation. As β-oxidation increases, the accumulated acetyl-CoA

acts as a negative regulator of pyruvate dehydrogenase (PDH) and limits the conversion of pyruvate to acetyl-CoA. Acetyl-CoA concurrently acts as a positive regulator of pyruvate carboxylase (PC), which facilitates the conversion of pyruvate to OAA and supports TCA cycle anaplerosis as well as gluconeogenesis. In the event of elevated and sustained gluconeogenesis, OAA availability would become limiting for citrate formation/TCA cycle activity and lead to the shunting of acetyl-CoA into ketogenesis, where coenzyme A (CoA) can be released and recycled to support β-oxidation. This sequence classically describes how acetyl-CoA is partitioned between the TCA cycle and ketogenesis over the transition from fed to fasting. Prior work using stable isotope tracers to interrogate these pathways and estimate hepatic β-oxidation in human subjects are presented.

NAFL threshold

5% Hepatic TG

2% Hepatic TG

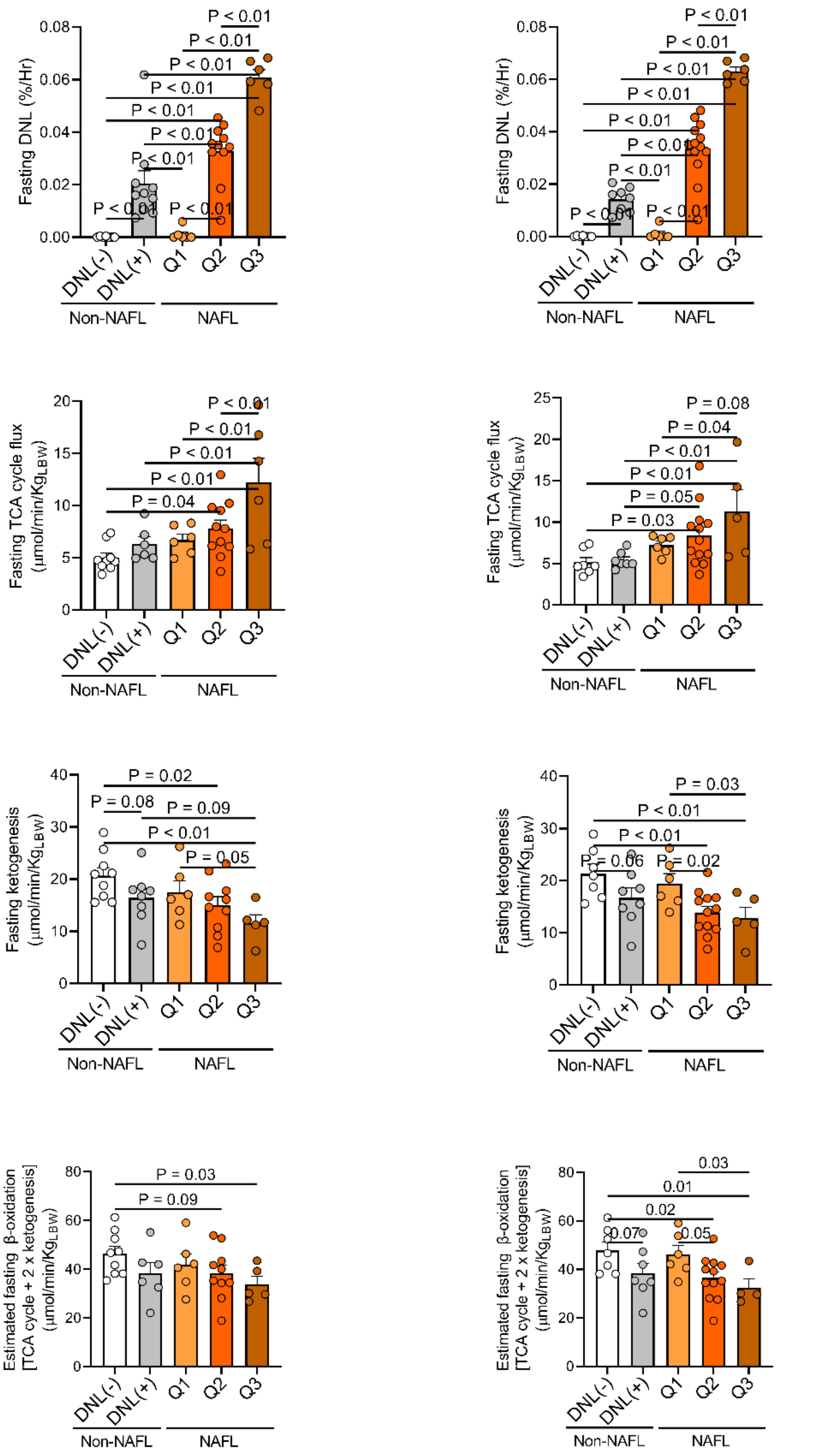

Supplemental Figure S2. A comparison of the groups studied using two different cut-offs for hepatic triglyceride (TG) content to define non-alcoholic fatty liver (NAFL) (≥5% and ≥2%). Hepatic TG content was determined by ^{1}H MRS. Using a cut-off for hepatic TG content of 2%, the distinctions between the groups are similar or amplified compared to a 5% cut-off. Using a 2% threshold for NAFL, the apparent rate of β-oxidation declined significantly across the NAFL quartiles of fasting de novo lipogenesis (DNL). *Abbreviations*: TCA, tricarboxylic acid; LBW, lean body weight. Statistical significance between groups was determined using a one-way ANOVA. Data are presented as mean ± SEM.

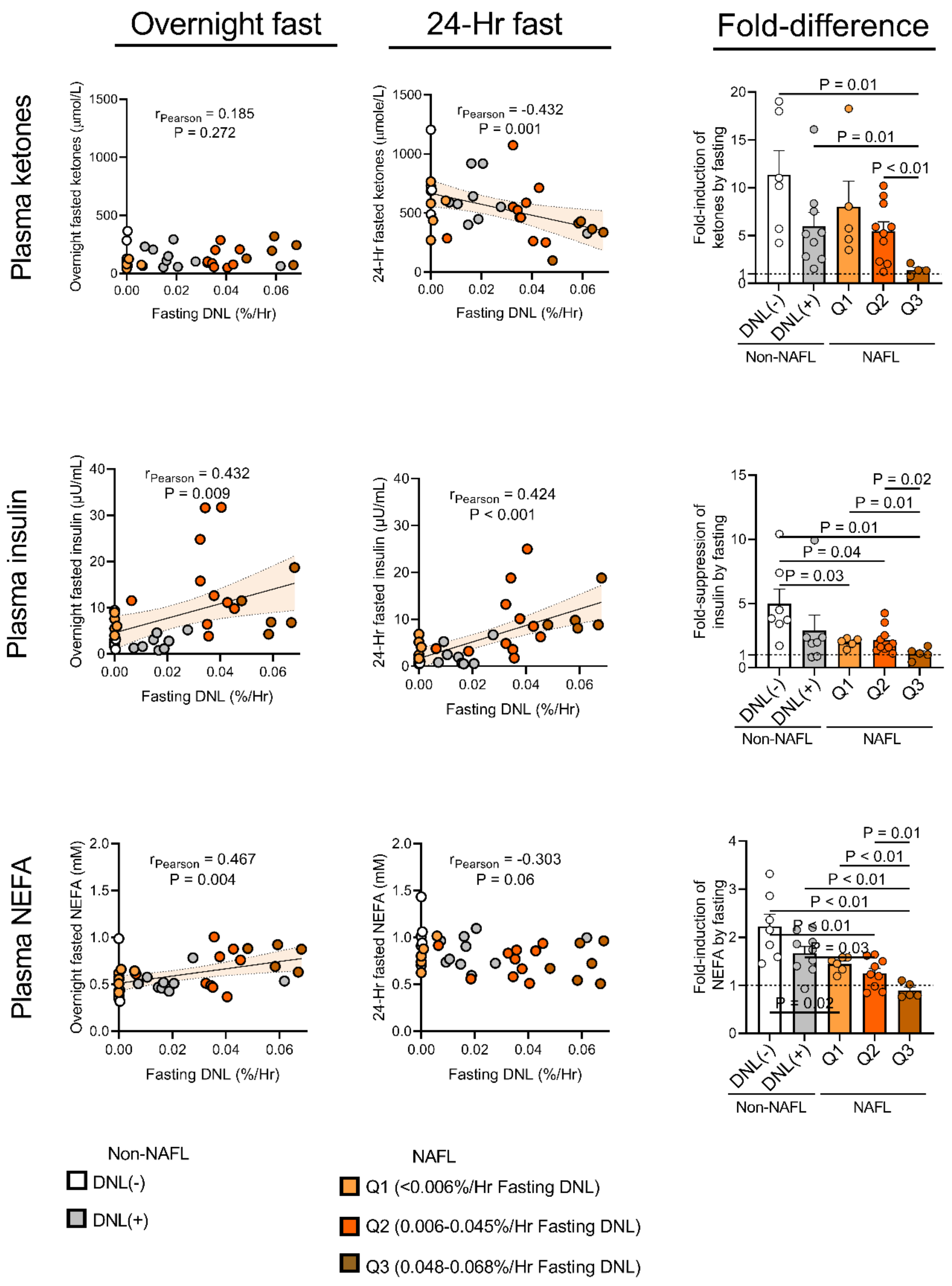

Supplemental Figure S3. Correlations between overnight and 24-hr fasted plasma ketone, insulin, and non-esterified fatty acid (NEFA) concentrations to 24-hr fasting de novo lipogenesis

(DNL) levels among the subjects studied. Pearson correlations and one-way ANOVA were used to test for statistical significance. Non-correlative data are presented as mean ± SEM.

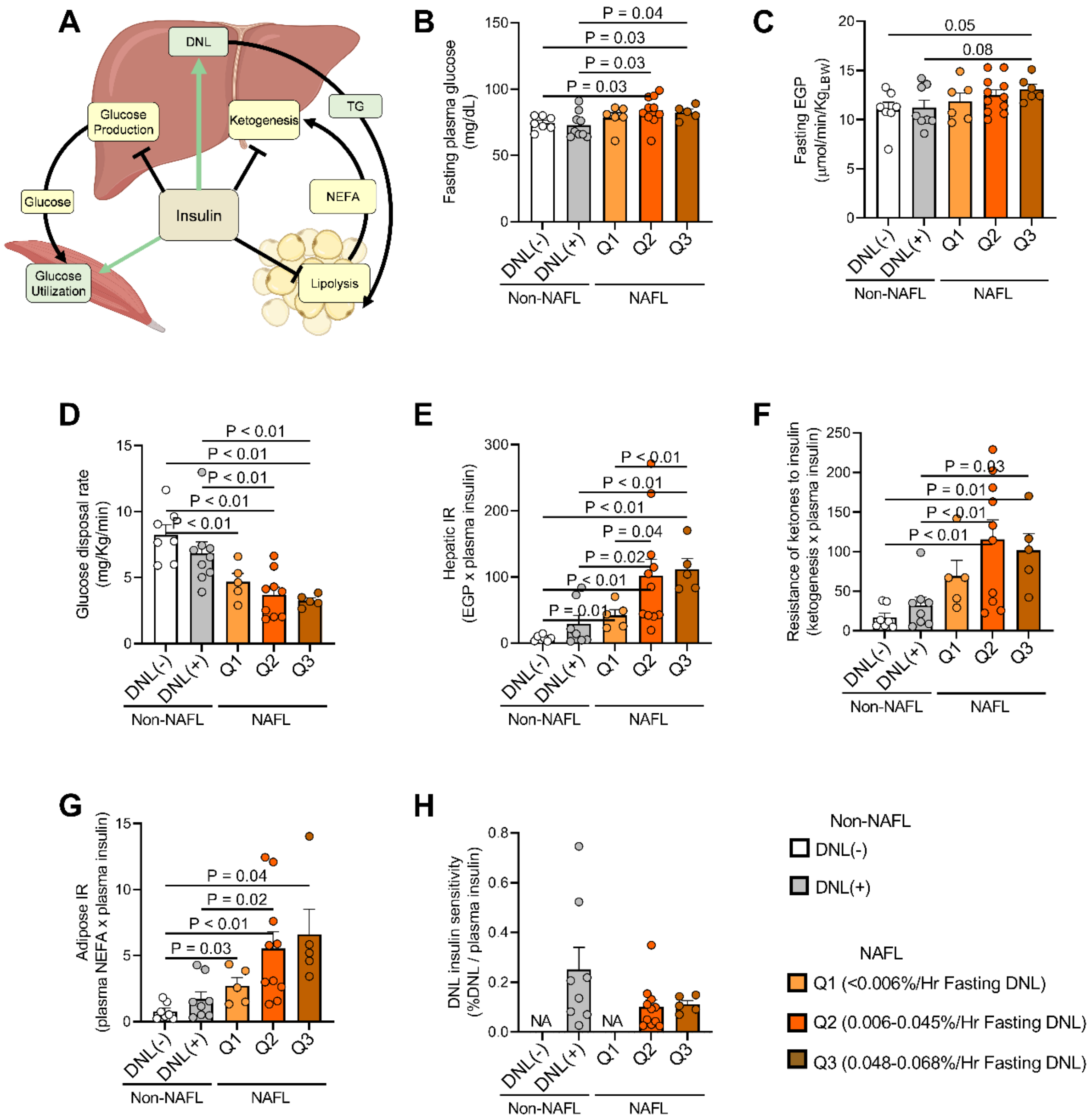

Supplemental Figure S4. (A) Under normal conditions, insulin suppresses adipose lipolysis, glucose production, and ketogenesis while promoting de novo lipogenesis (DNL) and peripheral glucose uptake by muscle and triglyceride (TG) by adipose tissue. **(B)** Fasting plasma glucose levels after a 24-hr fast were elevated among non-alcoholic fatty liver (NAFL) subjects with persistent lipogenic activity (Q2 and Q3). **(C)** Endogenous glucose production (EGP) after a 24-hr fast was elevated in the NAFL subjects with the highest level of persistent fasting DNL (Q3).

(**D-H**) Means of individual groups shown as correlations in **Figure 4**. *Abbreviations*: IR, insulin resistance. Statistical significance between groups was determined using a one-way ANOVA. Data are presented as mean ± SEM.

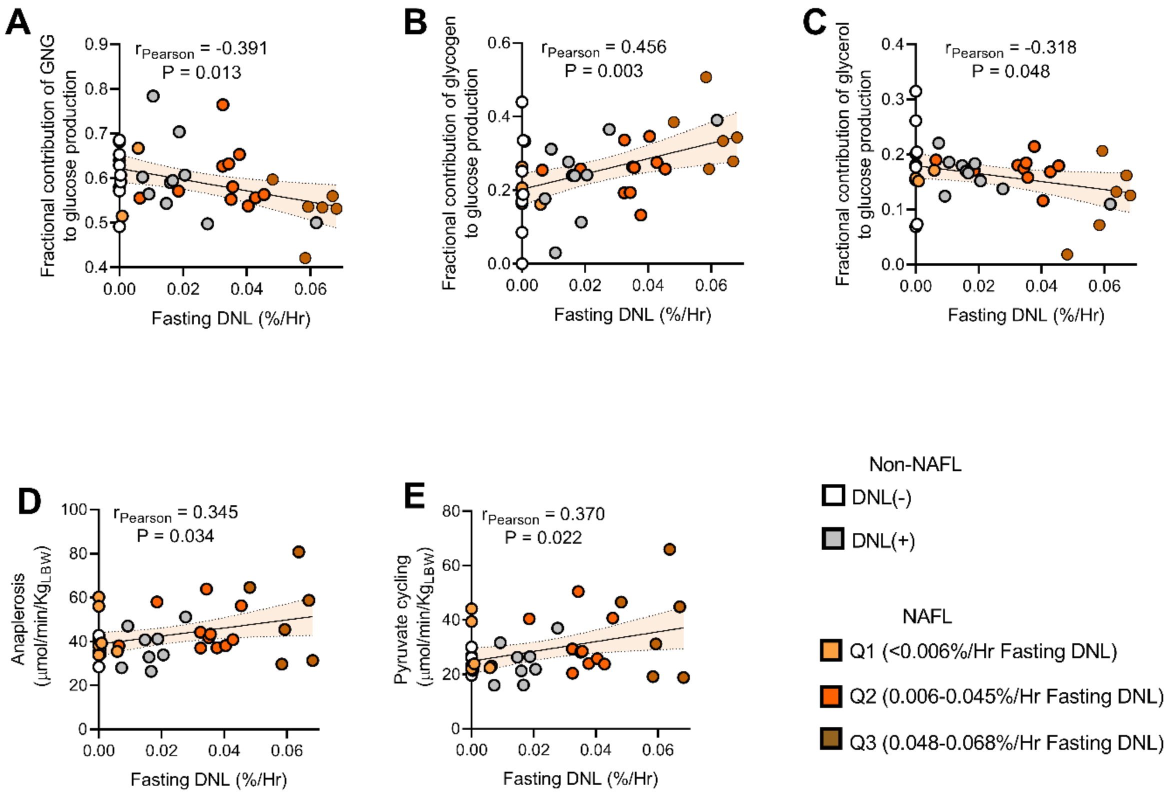

Supplemental Figure S5. Correlation between fasting de novo lipogenesis (DNL) levels and **(A)** fractional gluconeogenesis from tricarboxylic acid (TCA) cycle precursors, **(B)** fractional glycogenolysis, **(C)** fractional gluconeogenesis from glycerol, **(D)** TCA cycle anaplerosis, and **(E)** pyruvate cycling. All data was obtained after a 24-hr fast. Pearson correlations were used to test for statistically significant relationships.

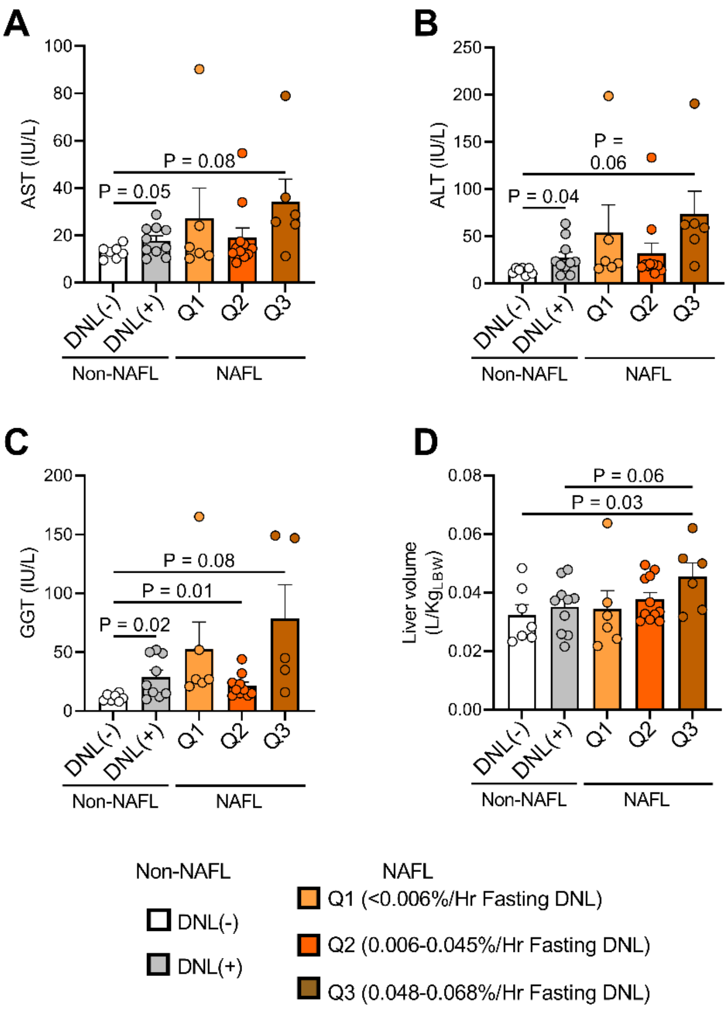

Supplemental Figure S6. (**A-D**) Means ± SEM of individual groups that were shown as correlations in **Figure 6**. *Abbreviations*: AST, aspartate aminotransferase; ALT, alanine aminotransferase; GGT, gamma glutamyl transferase, LBW, lean body weight. Statistical significance between groups was determined using a one-way ANOVA.

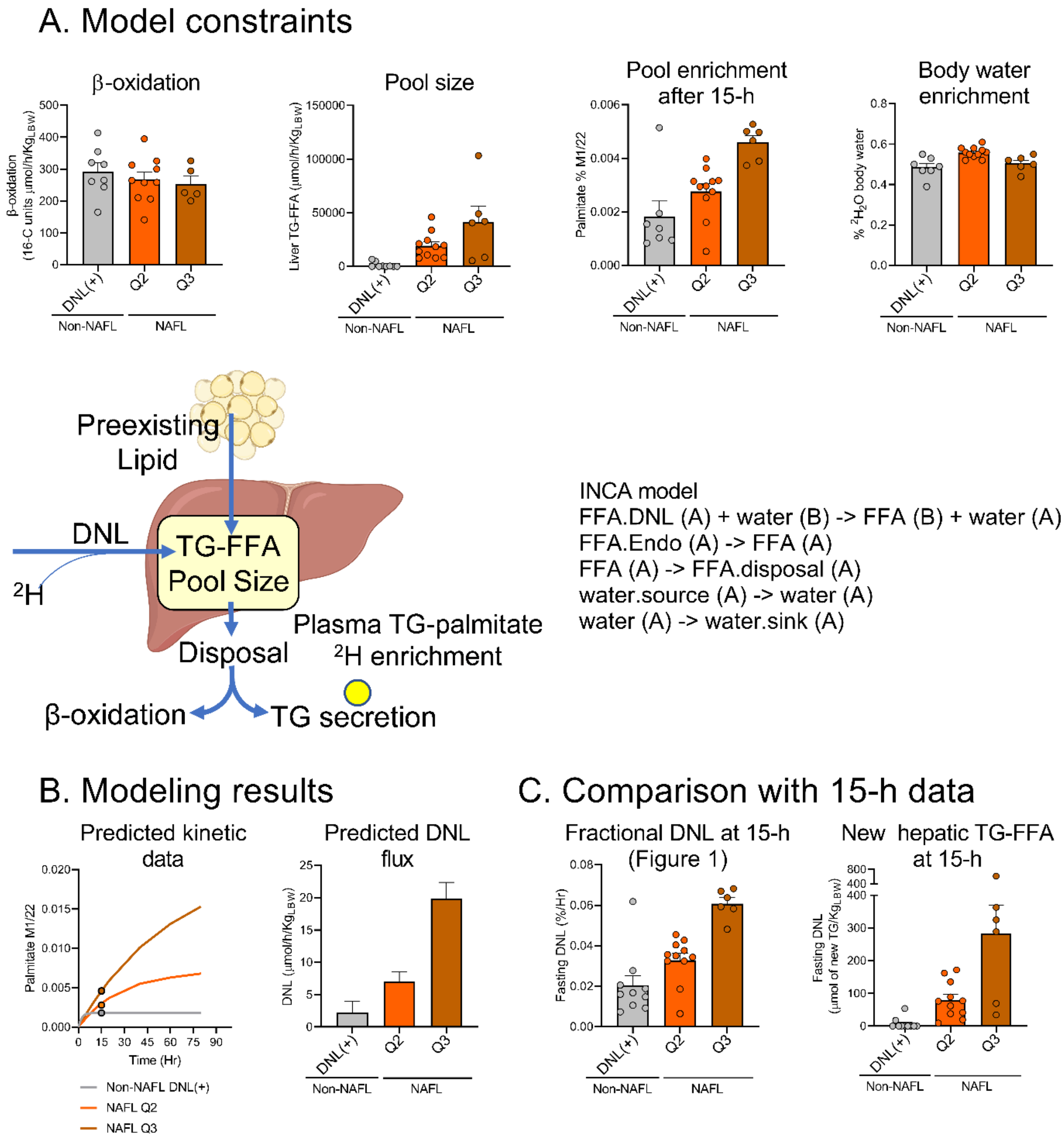

Supplemental Figure S7. Simulated hepatic triglyceride pool dynamics. (A) β-oxidation, as described in the main text but converted to μmol of 16 carbon units/h/Kg$_{LBW}$, was assumed to be 50% of the disposal rate, and triglyceride (TG) secretion was assumed to make up the other 50%. This value of TG-FFA secretion is similar to measured values in obese subjects after unit conversion (e.g., Lytle et al. J Clin Endocrinol Metab. 2019; and Heebøll et al., Diabetes 2022).

Hepatic TG pool size, expressed as μmol TG-FFA/ Kg_{LBW}, was estimated from the liver volume (**Supplemental Figure S6D**) and TG content (**Figure 1I**), using previously described assumptions (Szczepaniak et al. Am. J. Physiol., 1999). The 15-h pool enrichment was taken from the plasma TG-palmitate enrichment divided by 22 exchangeable hydrogens, and body water was measured from plasma. A turnover model in a single TG-FFA pool was simulated in INCA 2.0 (https://mfa.vueinnovations.com/). **(B)** Modeling TG kinetics resulted in estimates of palmitate enrichment dynamics and absolute rates of de novo lipogenesis (DNL). Notably, subjects without non-alcoholic fatty liver (NAFL) are predicted to be near steady-state, but NAFL subjects are predicted to be far from steady-state. **(C)** DNL flux in NAFL subjects measured using the predicted TG dynamics was enhanced relative to the 15-hr fractional DNL (%/Hr) reported in the main figures but followed a similar trend, especially when the data were expressed as DNL and liver TG-FFA content was used to estimate the absolute amount of new hepatic TG-FFA. *Abbreviations*: FFA, free fatty acid; LBW, lean body weight; INCA, isotopomer network compartmental analysis; Endo, endogenous.
